# Supplementary material for: Validation of urinary reproductive hormone measurements using a novel smartphone connected reader
Source: Sci Rep. 2023 Jun 7;13:9227. doi: 10.1038/s41598-023-36539-w (PMC10247788; doi:10.1038/s41598-023-36539-w)
Supplement: Supplementary file 1 — Supplementary Information. [file 41598_2023_36539_MOESM1_ESM.docx]

**Validation of urinary reproductive hormone measurements usinga novel smartphone connected reader**

**Siddharth Pattnaik^1*^, Dipankar Das^1^, Varun Akur Venkatesan^2^**

Siddharth Pattnaik^1*^, Dipankar Das^1^, Varun Akur Venkatesan^2^

^*^Corresponding author: siddharth@inito.com

^1^Samplytics Technologies Pvt. Ltd., 44, SKS Plaza, 2nd Floor, 100 Ft Road, Koramangala 4th Block, Bangalore 452012

^2^Inito Inc.,355 Bryant Street, Unit 403, San Francisco CA 94107

**Supplementary methods**

**Image processing and AI algorithms used in Inito Fertility Monitor:**

The Inito Fertility Monitor consists of two calibration blocks (shown in the blue box, supplementary figure 1) and the Inito test strip consists of a QR code and a detection window for reading the test and control lines. The appropriate focal length is calculated based on the focus level of the calibration blocks. The positions of the calibration blocks and the detection window are estimated using a basic template selection algorithm and patch validation (red dotted box, supplementary figure 1). The calibration blocks also contain pre-printed line intensities based on which the test line and control line intensities are estimated. The optical density (OD) for any sample is the ratio of the test and control lines normalized with respect to the background of the test strip itself (yellow boxes, supplementary figure 1). The OD is then mapped to the calibration curve generated using standard solutions (OD versus concentration) and the concentration is repredicted.


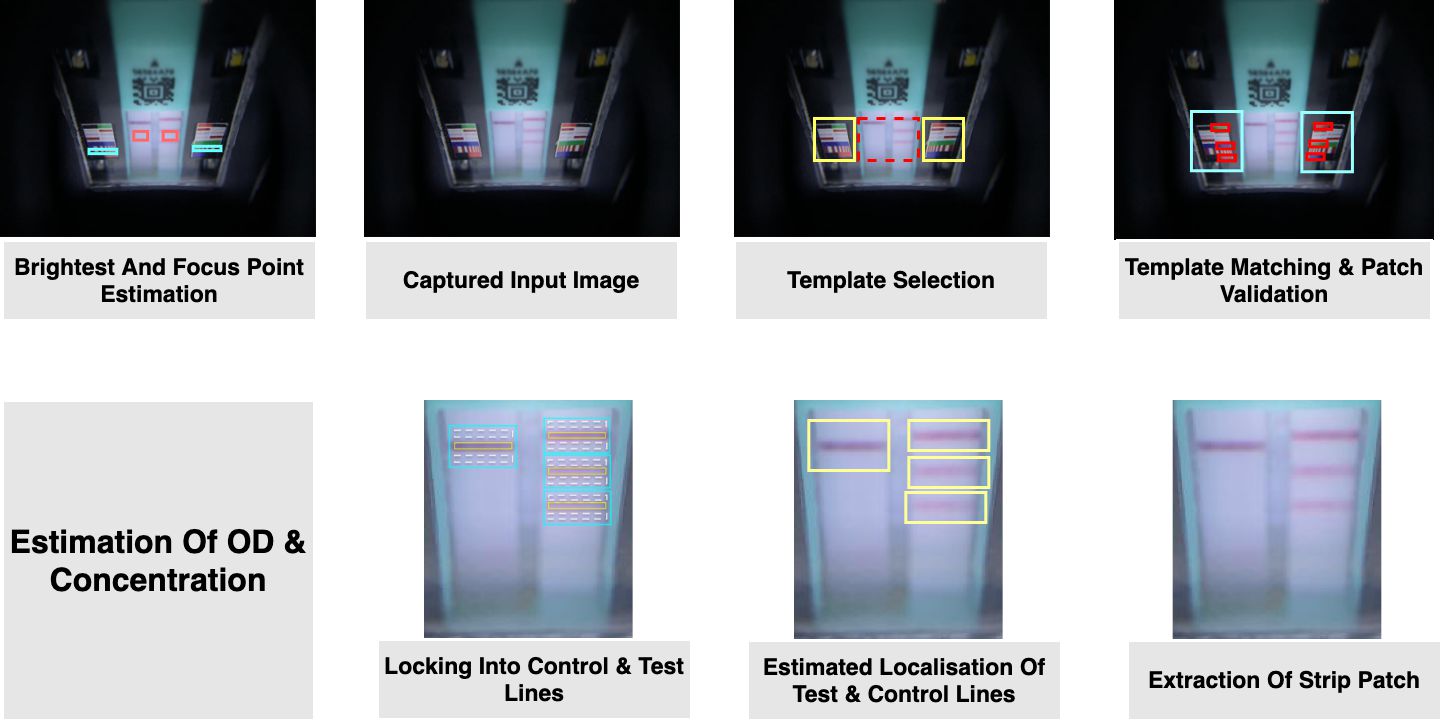


**Supplementary Figure 1**: Flowchart of image processing by the Inito Fertility Monitor showing the calibration blocks (bold blue rectangles), identification of detection zone by template matching (red dotted box), and identification of test and control lines for both the strips (yellow box, yellow dotted lines and blue rectangle).

While the imaging system can read test strips which are developed properly, certain handling errors may result in flow patterns on the test strip which may return inaccurate results. In order to avoid this, multiple AI algorithms are used to invalidate a test or to classify the type of error that has occurred. For instance, a common error observed when users take tests is the overdipped strip error, where excess fluid exposure causes the sample to reach the absorbent pad first, preventing the flow of fluid in the required direction (supplementary figure 2). To detect such errors, we train our system using Residual Neural Network (ResNet)^1^ where 10000 images are manually classified into two categories (normal and overdipped) and randomly separated into train images and test images in the ratio of 8:2. ResNet50 is then trained on the training images to classify them into normal or overdipped. The performance of the model is then checked using test images and if found satisfactory, the model is used to predict whether an image belongs to the normal category or overdipped category.


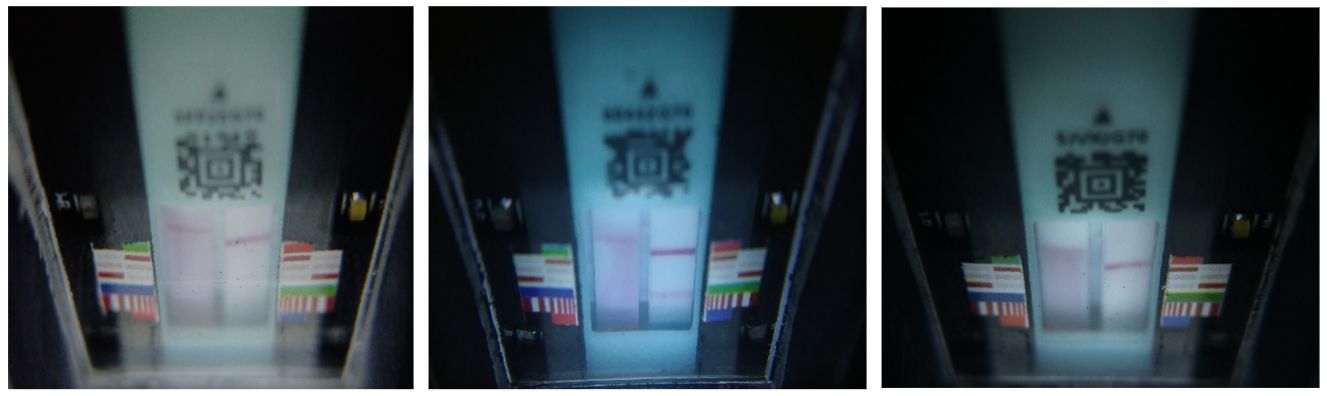


**Supplementary figure 2**: Representative images of overdipped assays

Similar to overdipped strip, there could be other issues with the test strip such as intrusion of foreign objects, defects in the test strip (speckles and breaks) and under-developed assays which are also identified in a similar. However, the efficacy of IFM in identifying these issues precisely and segregating them from normal images will be the scope of future publications.


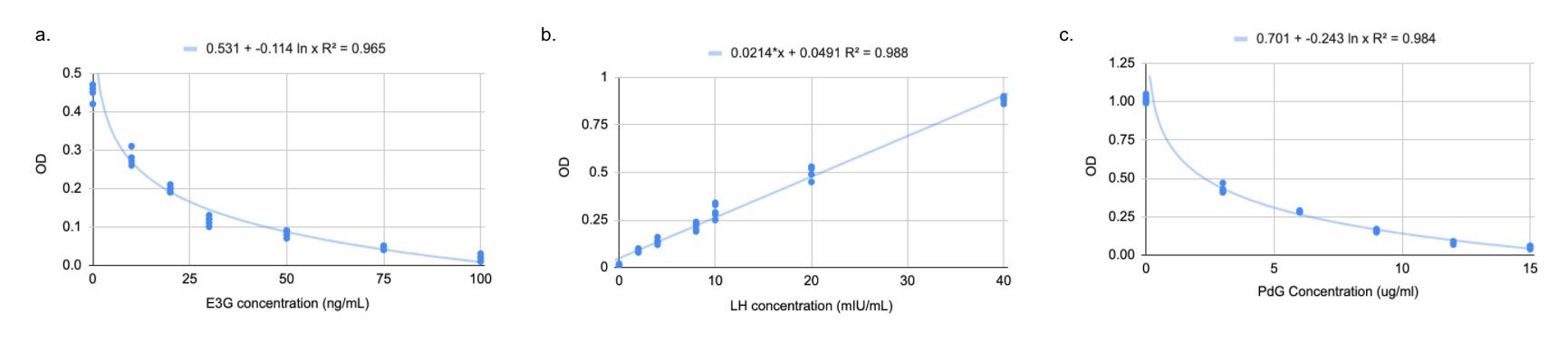


**Supplementary Figure 3**: OD to concentration curves used for reproducing E3G concentration (a), LH concentration (b) and PdG concentration (c) for all experiments performed


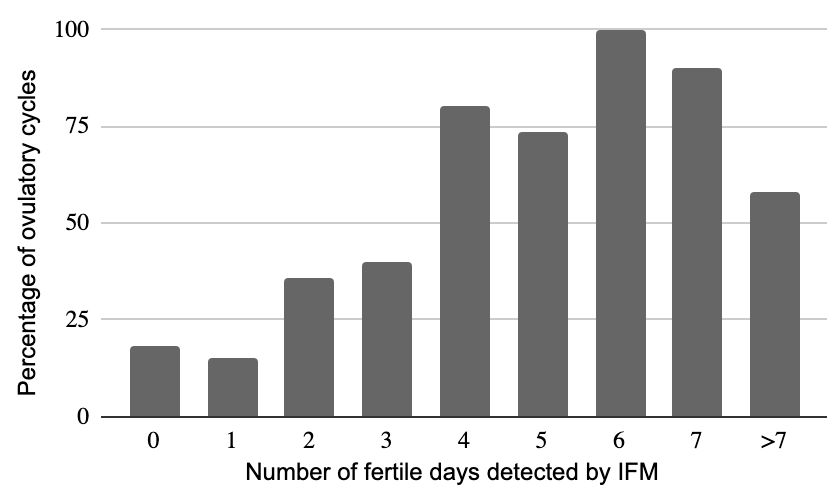


**Supplementary Figure 4**: Distribution of ovulatory cycles across number of high fertile days provided by Inito Fertility Monitor based on E3G rise.

**Supplementary tables**

**Supplementary Table.1. List of chemical standards from Sigma-Aldrich used in the evaluation of IFM along with the catalog numbers**

| **Chemical standard** | **Lot Number** |
| --- | --- |
| LH | L6420 |
| Progesterone | P0130 |
| Pregnanediol | 903620 |
| Estone-3-Glucuronide | E2127 |
| Acetaminophen | A3035 |
| Ascorbic Acid | A7506 |
| Caffeine | C0750 |
| Glucose | Y0001745 |
| Ampicillin | A9518 |
| Ketone | 179124 |
| Acetylsalicylic Acid | A3160 |
| Hemoglobin | ERMAD500 |
| Tetracycline | 31741 |
| Nitrite Positive | 1.06549 |
| Phenothiazine | 46624 |
| Ethanol | 02870 |
| Albumin | A2153 |

**Supplementary Table.2. Dilution protocol for preparing samples of E3G, PdG and LH in spiked male urine**

Stock concentration E3G: 1000ng/mL

Stock concentration LH: 1000mIU/mL

Stock concentration PdG: 50ug/mL

| **Nr** | **Target conc. E3G (ng/mL)** | **Target conc. LH**  **[mIU/ml]** | **Target conc. PdG[µg/ml]** | **E3G (µl)** | **LH (µl)** | **PdG (µl)** | **Spiked urine (µl)** |
| --- | --- | --- | --- | --- | --- | --- | --- |
| **1** | 0 | 0 | 0 | 0 | 0 | 0 | 2000 |
| **2** | 10 | 2 | 3 | 20 | 4 | 120 | 1756 |
| **3** | 20 | 4 | 6 | 40 | 8 | 240 | 1712 |
| **4** | 35 | 10 | 9 | 70 | 20 | 360 | 1550 |
| **5** | 50 | 20 | 12 | 100 | 40 | 480 | 1380 |
| **6** | 75 | 40 | 15 | 150 | 80 | 600 | 1170 |

**Supplementary Table.3. Inter-lot precision analysis of Inito fertility test strips at four concentrations of E3G:10, 35, 50 and 100ng/mL, PdG: 3, 6, 12 and 15ug/mL and LH: 2, 8, 10 and 40mIU/mL**

| **PdG conc** | **Re-predicted PdG conc** | **PdG CV** | **Spiked E3G conc** | **Re-predicted E3G conc** | **E3G CV** | **Spiked LH conc** | **Re-predicted LH conc** | **LH CV** |
| --- | --- | --- | --- | --- | --- | --- | --- | --- |
| 3 | 3.09 | 6.6% | 10 | 8.2 | 7.2% | 2 | 1.95 | 9.1% |
|  | 2.87 |  |  | 9.61 |  |  | 1.98 |  |
|  | 3.43 |  |  | 9.8 |  |  | 2.14 |  |
|  | 2.89 |  |  | 10.57 |  |  | 2.28 |  |
|  | 2.91 |  |  | 9.3 |  |  | 2.56 |  |
|  | 3.28 |  |  | 8.69 |  |  | 2.3 |  |
|  | 3.37 |  |  | 8.83 |  |  | 2.27 |  |
|  | 3.19 |  |  | 9.58 |  |  | 1.98 |  |
|  | 2.95 |  |  | 9.95 |  |  | 1.93 |  |
|  | 3.08 |  |  | 9.38 |  |  | 2.04 |  |
| 6 | 5.15 | 5.8% | 35 | 34.57 | 4.8% | 8 | 7.68 | 3.1% |
|  | 5.48 |  |  | 31.19 |  |  | 7.84 |  |
|  | 5.57 |  |  | 33.6 |  |  | 8.03 |  |
|  | 6.03 |  |  | 36.48 |  |  | 8.15 |  |
|  | 6.19 |  |  | 35.07 |  |  | 8.52 |  |
|  | 5.92 |  |  | 32.5 |  |  | 7.91 |  |
|  | 6.08 |  |  | 33.09 |  |  | 7.99 |  |
|  | 5.87 |  |  | 34.52 |  |  | 8.18 |  |
|  | 6.19 |  |  | 35.8 |  |  | 8.20 |  |
|  | 5.88 |  |  | 35.47 |  |  | 8.38 |  |
| 12 | 11.88 | 4.4% | 50 | 51.53 | 3.6% | 10 | 10.48 | 6.5% |
|  | 13.01 |  |  | 46.39 |  |  | 10.59 |  |
|  | 11.88 |  |  | 49.82 |  |  | 9.44 |  |
|  | 12.09 |  |  | 51.09 |  |  | 9.52 |  |
|  | 12.58 |  |  | 48.74 |  |  | 9.89 |  |
|  | 12.62 |  |  | 50.29 |  |  | 9.01 |  |
|  | 13.2 |  |  | 51.19 |  |  | 10.92 |  |
|  | 11.79 |  |  | 47.65 |  |  | 10.63 |  |
|  | 11.85 |  |  | 50.39 |  |  | 10.84 |  |
|  | 11.82 |  |  | 51.86 |  |  | 10.27 |  |
| 15 | 14.03 | 3.7% | 100 | 110.24 | 4.2% | 40 | 37.11 | 3.6% |
|  | 14.58 |  |  | 103.42 |  |  | 41.37 |  |
|  | 15.08 |  |  | 100.37 |  |  | 39.6 |  |
|  | 15.88 |  |  | 98.65 |  |  | 40.58 |  |
|  | 15.83 |  |  | 99.07 |  |  | 39.67 |  |
|  | 14.68 |  |  | 105.48 |  |  | 41.37 |  |
|  | 14.9 |  |  | 102.49 |  |  | 42.38 |  |
|  | 15.08 |  |  | 99.61 |  |  | 40.19 |  |
|  | 15.25 |  |  | 100.03 |  |  | 40.19 |  |
|  | 15.25 |  |  | 109.86 |  |  | 41.28 |  |

**Supplementary Table 4. Cross reactivity analysis of Inito Fertility test strips with different urine metabolites**

| **Interfering substance** | **Concentration** | **PdG test** | **E3G test** | **LH test** |
| --- | --- | --- | --- | --- |
| LH | 600mIU/mL | No | No | NA |
| hCG | 10000mIU/mL | No | No | No |
| Progesterone | 100ng/mL | No | No | No |
| Pregnanediol | 60ug/mL | NA | No | No |
| Estrone-3-glucuronide | 600ng/mL | No | NA | No |
| Ethanol | 1% | No | No | No |
| Albumin | 100mg/dL | No | No | No |
| Glucose | 2000mg/dL | No | No | No |
| Ketone | 1% | No | No | No |
| Ampicillin | 20mg/dL | No | No | No |

**Supplementary reference**

1. He, Kaiming, Xiangyu Zhang, Shaoqing Ren, and Jian Sun. "Deep residual learning for image recognition." In *Proceedings of the IEEE conference on computer vision and pattern recognition*, pp. 770-778. 2016.
